# Spaced Training Enhances Contextual Fear Memory *via* Activating Hippocampal 5-HT2A Receptors

**DOI:** 10.3389/fnmol.2019.00317

**Published:** 2020-01-24

**Authors:** Lizhu Jiang, Liping Wang, Yan Yin, Mengke Huo, Chao Liu, Qixin Zhou, Dafu Yu, Lin Xu, Rongrong Mao

**Affiliations:** ^1^Department of Neuropsychopathy, Clinical Medical School, Dali University, Dali, China; ^2^Key Lab of Animal Models and Human Disease Mechanisms of Chinese Academy of Sciences & Yunnan Province, Kunming Institute of Zoology, Kunming, China; ^3^Department of Central Laboratory, The Third People’s Hospital of Yunnan Province, Kunming, China; ^4^Key Lab of Animal Models and Human Disease Mechanisms of Chinese Academy of Sciences, University of the Chinese Academy of Sciences, Beijing, China; ^5^Department of Nuclear Medicine, The First People’s Hospital of Yunnan Province, Kunming, China; ^6^Department of Pathology and Pathophysiology, School of Basic Medical Science, Kunming Medical University, Kunming, China

**Keywords:** spacing effect, 5-HT2A receptor, Rac1, hippocampus, contextual fear memory

## Abstract

Spaced training is robustly superior to massed training, which is a well-documented phenomenon in humans and animals. However, the mechanisms underlying the spacing effect still remain unclear. We have reported previously that spacing training exerts memory-enhancing effects by inhibiting forgetting *via* decreasing hippocampal Rac1 activity. Here, using contextual fear conditioning in rat, we found that spaced but not massed training increased hippocampal 5-HT2A receptors’ expression. Furthermore, hippocampal administration of 5-HT2A receptor antagonist MDL11939 before spaced training blocked the enhanced memory, while hippocampal administration of 5-HT2A receptor agonist TCB-2 before massed training promoted the memory. Moreover, MDL11939 activated hippocampal Rac1, while TCB-2 decreased hippocampal Rac1 activity in naïve rats. These results indicated the possibility of interaction between 5-HT2A receptors and Rac1, which was demonstrated by co-immunoprecipitation experiments. Our study first demonstrates that activation of hippocampal 5-HT2A is a mechanism underlying the spacing effect, and forgetting related molecular Rac1 is engaged in this process through interacting with 5-HT2A receptors, which suggest a promising strategy to modulate abnormal learning in cognitive disorders.

## Introduction

Spaced training is more effective than massed training in producing long-term memory, which was first discovered by Ebbinghaus (1885) and called it the spacing effect. The spacing effect is highly conserved among species (Mauelshagen et al., 1998; Beck et al., 2000; Philips et al., 2013). Several molecules such as MAPK, CREB, and protein phosphatase 1 have been reported to modulate the spacing effect (Genoux et al., 2002; Pagani et al., 2009; Naqib et al., 2012; Smolen et al., 2016). Our previous study shows that spacing training improves contextual fear memory by inhibiting hippocampal Rac1 activity in rats (Jiang et al., 2016b). Further understanding the mechanisms underlying the spacing effect will provide insight into the modulation of learning and memory.

Serotonin (5-hydroxtryptamine, 5-HT) is a monoamine neurotransmitter widely distributed throughout the central nervous system, which is involved in learning and memory (Meneses and Liy-Salmeron, 2012; Meneses, 2013). There are at least 14 5-HT receptors divided into seven families (Pytliak et al., 2011), among which 5-HT2A receptors are highly expressed in the brain regions essential for learning and memory such as the hippocampus (Xu and Pandey, 2000; Meneses, 2002; Williams et al., 2002; Varnäs et al., 2004; Zhang and Stackman, 2015), which is required for the formation and retrieval of contextual fear memory (Phillips and LeDoux, 1992; Sanders et al., 2003). A previous study shows that activation of 5-HT2A receptors in the basolateral amygdala improves the acquisition of conditioned defeat (Clinard et al., 2015). Administration of 5-HT2A receptor agonist TCB-2 is also found to facilitate consolidation and extinction of fear memory (Zhang et al., 2013). Although these results support the view that activation of 5-HT2A receptor may enhance memory, whether 5-HT2A receptors improve memory by regulating the spacing effect is still unknown.

Rac1, a small GTPase, can regulate the forgetting of memory (Shuai et al., 2010; Liu et al., 2018) and synaptic plasticity (Martinez and Tejada-Simon, 2011; Golden et al., 2013). Our recent investigations report that activation of hippocampal Rac1 promotes the forgetting of contextual fear memory in spaced learning rats, while inhibition of hippocampal Rac1 activity prevents the forgetting of contextual fear memory in massed learning rats (Jiang et al., 2016b). Furthermore, we find that regulation of hippocampal Rac1 activity can also alter the effects of massed and spaced extinction training (Jiang et al., 2016a). These results indicate that hippocampal Rac1 participates in the spacing effect. It is noted that stimulation of 5-HT2A receptors can activate Rac1 *via* TGase *in vitro* (Dai Y. et al., 2008), which indicates a possibility of interaction between 5-HT2A receptors and Rac1. Furthermore, hippocampal 5-HT transporters are downregulated in forgetting of associative memory (Tellez et al., 2012). However, it is unknown whether Rac1-dependent forgetting is involved in the mechanisms of memory enhancement induced by the 5-HT2A receptor activation.

We therefore hypothesize that hippocampal 5-HT2A receptors may be involved in the spacing effect. In the present study, we first investigated the expression patterns of hippocampal 5-HT2A receptors separately in spaced and massed training groups in the contextual fear conditioning and then used pharmacological approaches to activate or inhibit hippocampal 5-HT2A receptors to modulate the spacing effect. Furthermore, we also investigated whether Rac1-dependent forgetting is engaged in the effect of 5-HT2A receptors on the spacing effect. We investigated these questions by using a combination of immunoblotting, immunohistochemical assays, and Co-immunoprecipitation and behavioral tests.

## Materials and Methods

### Animals

Male Sprague–Dawley rats (inbred strain, Animal House Center, Kunming General Hospital, Kunming) weighing 200–250 g were group-housed (five per cage) in a thermoregulated environment with a 12 h light/dark cycle and lights on at 8:00 am. Rats were given *ad libitum* access to water and food. All experiments were carried out between 09:00 and 17:00. Experimental protocols were approved by the animal ethics committee of Kunming Institute of Zoology, Chinese Academy of Sciences.

### Contextual Fear Conditioning

The animals were placed into chambers (30 × 24 × 21 cm^3^, MED Associates, St. Albans, VT, USA) for a 2 min free exploration to establish baseline freezing, and then rats received five footshocks (0.8 mA, 1 s duration). The five trials were presented at different inter-trial intervals (ITIs): 12 s (massed training) and 122 s (spaced training; Jiang et al., 2016b). Rats were removed from the conditioning chamber and returned to their home cages 12 min later. Twenty-four hours after training, contextual memory was tested for 5 min. Freezing was tracked and analyzed using Video Freeze Software (Med Associates). Percent freezing time to each ITI was presented to measure the strength of fear memory. In the massed training group, due to the short ITI (12 s) and rats always jumping immediately after suffering footshock, freezing time can only be scored at the baseline and after the fifth footshock.

### Cannula Implantation and Drug Infusion

Under phenobarbital sodium anesthesia, bilateral guide cannulas were implanted in the CA1 area of the dorsal hippocampus using the stereotaxic coordinates: anteroposterior (AP) −3.5 mm, mediolateral (ML) ± 2.5 mm, and dorsoventral (DV) −2.5 mm, according to the atlas of Paxinos and Watson (1998). The guide cannulas were fixed to the skull with dental acrylic. The rats were allowed to recover from surgery for 7 days. Immediately before fear conditioning, 5-HT2A antagonist MLD11939 (17 mmol, 1 μl/side, Tocris Bioscience) was dissolved in saline with 1% acetic acid and 10% sodium hydroxide (pH = 5.5; Clinard et al., 2015) and injected into the bilateral hippocampus of the spaced training group. Forty minutes before fear conditioning, 5-HT2A agonist TCB-2 (40 mmol, 1 μl/side, Tocris Bioscience) was dissolved in saline with 10% DMSO and injected into the bilateral hippocampus of the massed training group. All vehicle groups received the same volume of the vehicle. The microinjectors were left in place for an additional 1 min after infusion to allow the solution to diffuse away from the cannula tip.

### Immunohistochemical Staining

Rats were euthanized with Euthasol euthanasia solution and transcardially perfused with ice-cold 4% paraformaldehyde in 0.1 M phosphate-buffered saline (PBS). Brains were postfixed for 4 h at 4°C and cryoprotected in 30% sucrose in 0.1 M PBS. Serial coronal sections throughout the hippocampus were cut at a 40 μm thickness using a cryostat and stored in PBS. The sections were incubated with blocking buffer (5% bovine serum albumin, 0.3% Triton X-100 and PBS) for 1 h followed by overnight incubation with mouse anti-5-HT2A receptor antibody (1:500, Cat. PC176, Millipore) at 4°C. The sections were rinsed and transferred to Cy3-conjugated anti-Rabbit immunoglobulin G (IgG; 1:500, Cat. 111-165-144, Jackson ImmunoResearch) for 2 h at room temperature. The sections were examined using a confocal laser-scanning microscope (FV-1000, Olympus, Tokyo, Japan).

### Immunohistochemistry Data Analysis

Immunohistochemical staining of 5-HT2a receptor was quantified by pixel density. The CA1 area was the ROI for intensity analysis set. Images were corrected for variability in staining by calibrating based on highest and lowest (backgroud) density of staining between experimental groups. The back ground intensity was based on the optic intensity of vascular lumen in the section. Two coronal brain sections per animal were quantified at two different Bregma levels (−3.24 mm, −3.36 mm), and quantitative analyses were obtained at 200× magnification.

### Rac1 Activity Assay

The active form of Rac1 was bound to GTP, which was determined using a Rac1 pull-down activation assay for Rac1-GTP. Briefly, hippocampal tissues were homogenized in Mg^2+^ lysis buffer in the presence of protease and phosphatase inhibitors and centrifuged at 12,000× *g* for 15 min at 4°C. After preclearing the protein with A/G agarose beads (20 μl, Cat. 30301, Neweast), GTP-bound Rac1 was immunoprecipitated from the cell lysates using an anti-active Rac1 monoclonal antibody (1 μl, Cat. 26903, Neweast). Rac1-GTP as well as total Rac1 in the lysates, was examined by western blotting using a rabbit anti-Rac polyclonal antibody (1:300, Cat. 26005, Neweast).

### Co-immunoprecipitation Assay

Rat hippocampus was frozen in liquid nitrogen and homogenized in radio immunoprecipitation assay (RIPA) lysis buffer (Cat. P0013K, Beyotime) added with 1 mM PMSF, which immunoprecipitated with/without 2 μl anti-Rac1 antibody (Cat. 26903, Neweast) and 20 μl A/G agarose beads (20 μl, Cat. 30301, Neweast) or 2 μl nonspecific IgG antibodies (Cat. 2026, Santa Cruz). The co-immunoprecipitated proteins were identified by western blotting using a mouse anti-5-HT2A receptor antibody (1:2,000, Cat. MABN1595, Millipore).

### Western Blot Analyses

Rat hippocampus was frozen in liquid nitrogen and homogenized in RIPA buffer (Beyotime Biotech) added with 1 mmol PMSF. Samples were mixed (3:1) with the 4× SDS loading buffer [250 mmol Tris-Hcl, pH 6.8, 20% *β*-mercaptoethanol, 4% SDS, 0.004% bromophenol blue (wt/vol), 40% (vol/vol) glycerol], and denatured by boiling for 5 min at 100°C. Each sample was run on a SDS-PAGE (Bio-Rad) and transferred to a PVDF membrane. Blots were blocked at room temperature with block buffer (Cat. 820473, Millipore). For western analysis, we used rabbit polyclonal 5-HT2A receptor antibody (1:2,000, Cat. PC176, Millipore) and mouse monoclonal Rac1 antibody (1:300, Cat. 26003, New East), and mouse monoclonal GAPDH antibody (1:20,000, Cat. KC-5G5, Aksomics). Immunoreactivity was detected using luminata crescendo western HRP substrate (Cat. WBLUF0500, Millipore). The intensities of the detected bands in the western blots were quantified using ImageJ software.

### Data Analysis and Statistics

All data were expressed as means ± standard error (mean ± SEM). Data were analyzed by unpaired *t*-test or repeated analysis of variance, and between-group comparisons were made by one-way ANOVA followed by the least significant difference (LSD) test. Levene’s test of equality of variance was used to test the equality of variance (SPSS 16.0). Significance level was set at *p* < 0.05.

## Results

### Spaced Training Produces Stronger Fear Memory and Increases 5-HT2A Receptor Expression in the Hippocampus

**Figure 1:**
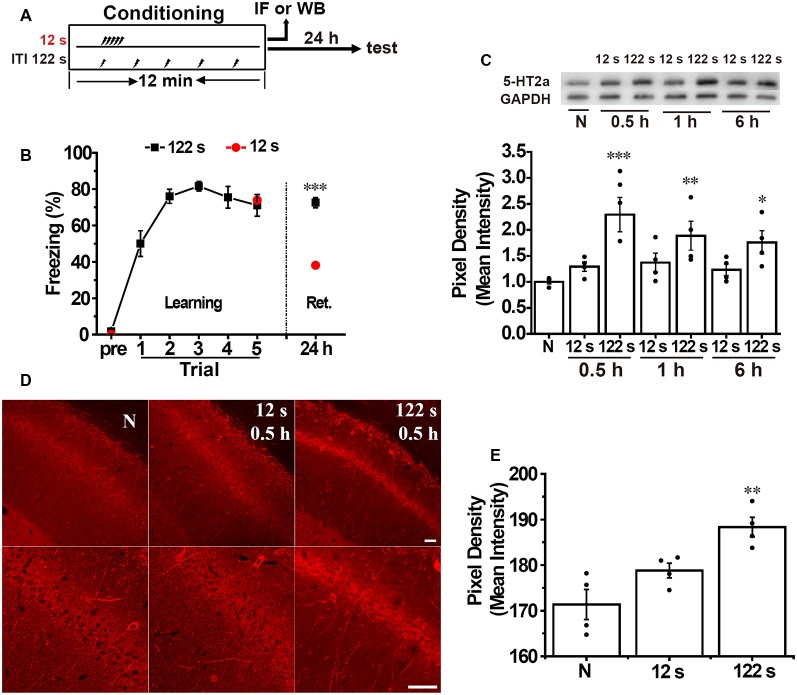
Spaced training enhances hippocampal 5-HT2A receptor expression and contextual fear memory. **(A)** Diagram for experimental procedures. **(B)** The spaced training group (122 s) showed a heightened contextual fear memory compared with the massed training group (12 s) at 24 h after training (*n* = 10 for each group). **(C)** Western blotting analysis showed that spaced but not massed training increased hippocampal 5-HT2A receptor expression at 30 min (0.5 h), 1 h, and 6 h after contextual fear conditioning compared with the naïve group (*n* = 4 for each group). **(D)** The 5-HT2A receptor positive staining in the hippocampal CA1 area significantly increased in the spaced but not in the massed group at 0.5 h after contextual fear conditioning [*p* < 0.05, **(E)**]. Scale bar = 50 μm. **(E)** Bar graph of (**D**; *n* = 4 for each group). All results were presented as mean ± SEM. *p < 0.05, **p < 0.01, ***p < 0.001.

Our previous study shows that spaced training enhances contextual fear memory. Rats received five footshocks with ITI of 12 s in the massed training group and 122 s in the spaced training group and 600 s in the long-spaced training group, and we found that the spaced and the long-spaced group presented higher freezing scores. Here, we used similar protocol as in the previous study (Figure 1A). Twenty-four hours following fear conditioning, the memory performance of the spaced training group (122 s) was significantly higher compared with the massed group (12 s; unpaired *t*-test, *t* = 5.478, *p* < 0.001, *n* = 10, Figure 1B).

Next, we measured the expression of 5-HT2A receptors in the hippocampus separately at 30 min (0.5 h), 1 h, and 6 h after contextual fear conditioning. We found that the levels of 5-HT2A receptors in the hippocampus were significantly different among the groups (one-way ANOVA, *F*_(6,21)_ = 4.844, *p* < 0.05, *n* = 4). Two-way ANOVA disclosed no significant group and time effects on the expression of 5-HT2A receptors (group × time effect: *F* = 0.791, *p* = 0.469). The level of hippocampal 5-HT2A receptors increased significantly in the spaced group at 0.5 h (*p* < 0.001), 1 h (*p* < 0.01), and 6 h (*p* < 0.05) after contextual fear training, while there were no significant alterations in the massed group at 0.5 h, 1 h, and 6 h time points (Figure 1C). Immunofluorescence staining showed that 5-HT2A receptors appeared to be preferentially localized to soma and dendrites. There were significant differences among groups (*F*_(2,9)_ = 11.920, *p* < 0.05, *n* = 4). Thirty minutes after fear conditioning, the pixel density of 5-HT2A receptors’ positive staining in the CA1 area was significantly enhanced in the spaced group (*p* < 0.01, Figure 1D) but not in the massed group (Figure 1E) compared with naive group. These results demonstrated that the spacing but not massed training increased the expression of hippocampal 5-HT2A receptors in contextual fear conditioning, which indicated that the activation of hippocampal 5-HT2A receptors may be involved in the spacing effect.

### Inhibition of Hippocampal 5-HT2A Receptors Blocks While Activation of Hippocampal 5-HT2A Receptors Promotes the Spacing Effect in Contextual Fear Conditioning

**Figure 2:**
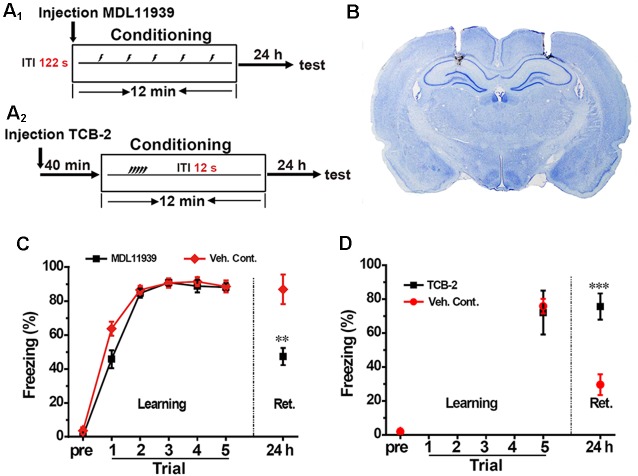
Inhibition of hippocampal 5-HT2A receptors blocked the spacing effect, while activation of hippocampal 5-HT2A receptors promoted the spacing effect. **(A_1_,A_2_)** Diagram for experimental procedures. **(B)** Representative Nissl staining confirming the implantation sites of the guide cannulas. **(C)** Hippocampal administration of 5-HT2A receptor antagonist MDL11939 blocked the enhanced fear memory in the spaced training (*n* = 8). **(D)** Hippocampal administration of 5-HT2A receptor agonist TCB-2 enhanced fear memory in the massed training (*n* = 9). All results were presented as mean ± SEM, **p < 0.01, ***p < 0.001.

We aimed to confirm whether the activation of hippocampal 5-HT2A receptors are involved in the spacing effect in contextual conditioning. First, we infused 5-HT2A receptor antagonist MDL119939 (17 mmol, 1 μl/side, MDL) into the bilateral hippocampus immediately before spaced training (Figure 2A_1_); the injection sites were confirmed by *postmortem* examination (Figure 2B). Twenty-four hours after contextual fear conditioning, freezing score was significantly decreased in the MDL group compared with the vehicle group (unpaired *t-test*, *t* = 3.741, *p* < 0.01, *n* = 8, Figure 2C). These results indicated that the spacing training exerts memory enhancement effects through the activation of hippocampal 5-HT2A receptors.

Next, we injected the 5-HT2A receptor agonist TCB-2 (40 mM, 1 μl/side) into the bilateral hippocampus 40 min before massed training (Figure 2A_2_). The learning curves showed that TCB-2 had no effect on learning. Twenty-four hours after contextual fear conditioning, freezing score was significantly higher in the TCB-2 group compared with the vehicle group (unpaired *t*-test, *t* = 6.242, *p* < 0.001, *n* = 9, Figure 2D). These results demonstrated that activation of hippocampal 5-HT2A receptors enhanced memory in the massed training rats.

In summary, inhibition of hippocampal 5-HT2A receptors blocked memory enhancement in spaced training, while activation of hippocampal 5-HT2A receptors enhanced memory in massed training, which indicated that the activation of hippocampal 5-HT2A receptors were engaged in the spacing effect in contextual fear conditioning.

### Inhibition of 5-HT2A Receptors Activates Rac1, While Activation of 5-HT2A Receptors Inhibits Rac1 Activity in the Hippocampus

**Figure 3:**
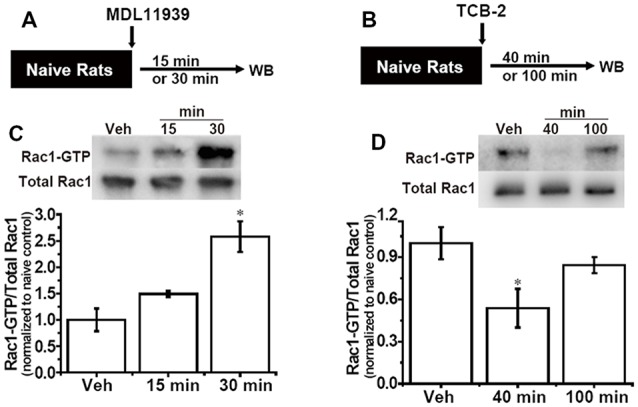
Inhibition of hippocampal 5-HT2A receptors activates Rac1 activity, activation of hippocampal 5-HT2A receptors inhibits Rac1 activity. **(A,B)** Diagrams of experimental procedures. **(C)** Hippocampal administration of 5-HT2A receptor antagonist MDL11939 increased Rac1 activity 30 min after administration. **(D)** Hippocampal administration of 5-HT2A receptor agonist TCB-2 inhibited Rac1 activity 40 min after administration. *n* = 4 for each group. All results were presented as mean ± SEM, *p < 0.05.

Our previous study indicated that hippocampal Rac1 activity regulated the spacing effect in the contextual fear conditioning. The above results showed that hippocampal 5-HT2A receptors also regulated the spacing effect. Moreover, evidence *in vitro* shows that stimulation of 5-HT2A receptors can alter the activity of Rac1. Thus, we next aimed to investigate whether Rac1-dependent forgetting is engaged in the effect of 5-HT2A receptors on the spacing effect. The 5-HT2A receptor antagonist MLD11939 (17 mmol, 1 μl/side) was administrated into the bilateral hippocampus of the naive rats; 15 min or 30 min later, the rats were sacrificed and hippocampal Rac1 activity was detected (Figure 3A). In a preliminary experiment rats were sacrificed 40 or 100 min later, but the activation of hippocampal Rac1 was not detected (S F2). Results showed that there were significant differences among groups (*F*_(2,9)_ = 9.698, *p* < 0.01, *n* = 4). MLD11939 slightly activated Rac1 15 min later and significantly promoted Rac1-GTP 30 min (*p* < 0.01) later (Figure 3C). Next, the 5-HT2A receptor agonist TCB-2 (40 mM, 1 μl/side) was administrated into the bilateral hippocampus of the naive rats, and hippocampal Rac1 activity was detected 40 min or 100 min after administration. The levels of Rac1-GTP were significantly different among groups (*F*_(2,9)_ = 38.329, *p* < 0.001, *n* = 4, Figure 3B). TCB-2 significantly inhibited hippocampal Rac1 activity 40 min after administration (*p* < 0.001) compared with the vehicle group, and this inhibition disappeared 100 min after TCB-2 administration (Figure 3D). These results demonstrated that Rac1-dependent forgetting was engaged in the effect of 5-HT2A receptors on the spacing effect.

### Hippocampal 5-HT2A Receptors Interact With Rac1

**Figure 4:**
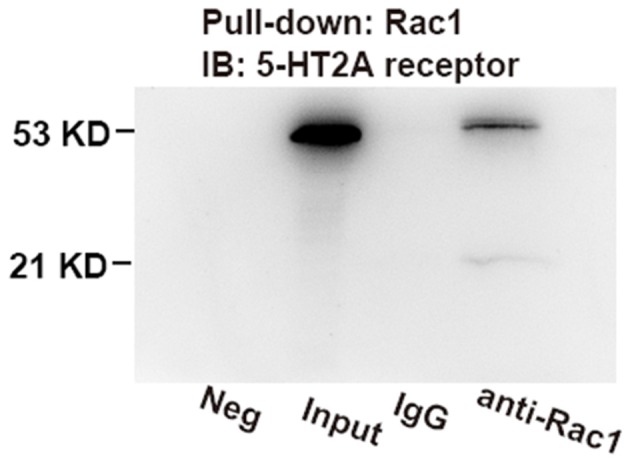
Co-immunoprecipitation (IP) assays demonstrated the interactions between hippocampal 5-HT2A receptors and Rac1. Rac1 was pulled down using anti-Rac1 antibody; 5-HT2A receptors were measured on immunoblots. Bands at 53 kDa were detected in input and Rac1 pull-down lane, which were not detected in the immunoglobulin G (IgG) lane, and a weak positive band around 21 kD was detected in the Rac1 lane.

The above result showed that Rac1-dependent forgetting was engaged in the effect of 5-HT2A receptors on the spacing effect; we wanted to further investigate whether 5-HT2A receptors interact with Rac1. We used an immunoprecipitated protein with anti-Rac1 antibody from the hippocampus of contextual fear learning rats and detected 5-HT2A receptor. As shown in Figure 4, 5-HT2A receptor was tested in Rac1 pull-down protein. The result demonstrated that there were interactions between Rac1 and 5-HT2A receptors. We also detected a weak positive band around 21 KD in the Rac1 lane after staining for 5-HT2A, which may be the Rac1-5-HT2A receptor compounds.

## Discussion

In the present study, we demonstrate that the activation of hippocampal 5-HT2A receptors is involved in the spacing effect in contextual fear conditioning, and Rac1-dependent forgetting is engaged in the effect of 5-HT2A receptors on the spacing effect. We further demonstrate that there is an interaction between hippocampal 5-HT2A receptors and Rac1. To our knowledge, we first provide evidence that activation of hippocampal 5-HT2A is a mechanism underlying the spacing effect and forgetting related molecular Rac1 engaged in this process through interacting with 5-HT2A receptors.

5-HT2A receptors belonging to the GPCR family are widely distributed in the neocortex, amygdala, and hippocampus of rats, primates, and humans (Jakab and Goldman-Rakic, 1998). Central 5-HT2A receptors exert diverse physiological, behavioral, psychological functions including emotional memory (Zhang and Stackman, 2015). Studies have demonstrated that 5-HT2A receptors play an important role in fear learning. Intra-peritoneal injection of 5-HT2A receptor agonist TCB-2 following fear conditioning enhanced fear memory in mice (Zhang et al., 2013). Administration of 5-HT2A receptor antagonist MDL11939 into the basolateral amygdala impaired, while 5-HT2A receptor agonist TCB-2 promoted the acquisition of conditioned defeat in Syrian hamsters (Clinard et al., 2015). These studies implicate that the activation of central 5-HT2A receptors enhances fear memory. However, the signaling pathways downstream from 5-HT2A receptors remain unclear. The present study found that Rac1 is a novel downstream target of 5-HT2A receptors.

Rac1 is a member of the Rho family of small GTPases, which play a crucial role in synapse plasticity (Oh et al., 2010) and learning and memory (Diana et al., 2007; Gao et al., 2015). Our previous study found that hippocampal Rac1 activity was inhibited in the spacing training group (Jiang et al., 2016b). In the previous study, rats received five footshocks with 12 s ITI in the massed group, and 122 s ITI in the spaced group and 600 s ITI in the long-spaced group. Spaced but not massed training inhibited hippocampal Rac1 activity and enhanced contextual fear memory. Consistent with this result, activation of hippocampal Rac1 by Rac1 activator weakened the fear memory in spaced training rats while inhibition of hippocampal Rac1 by Rac1 inhibitor promoted fear memory in massed training rats. Here we found that 5-HT2A receptors also regulated the spacing effect, and Rac1-dependent forgetting was engaged in the 5-HT2A effect on the spacing effect through interactions between 5-HT2A receptors and Rac1. Previous study showed that stimulation of 5-HT2A receptors activated Rac1 *via* TGase *in vitro* (Dai Y. et al., 2008). Contrary to this result, we found that stimulation of hippocampal 5-HT2A receptors inhibited Rac1 activity, *vice versa*, inhibition of 5-HT2A receptors activated Rac1. The difference between our study and a previous study may be attributed to different models and 5-HT2A receptor agonist used. Dai Y. et al. (2008) used DOI, a 5-HT2A/2C receptor agonist in culture, while we used TCB-2, a 5-HT2A receptor agonist in the rat’s hippocampus. The Rho GTPases are activated by guanine nucleotide exchange (GEF) and deactivated by GTPase activating proteins. Kalirin-7 is a brain-specific GEF for the small GTPase Rac. Jones et al. (2009) found that the 5-HT2A receptor rapidly altered spine morphology through kaliren-7 signaling in the cortical pyramidal neurons. Here we found that hippocampal 5-HT2A receptors were upregulated 30 min after spaced training, and 5-HT2A receptor antagonist MDL11939 significantly activated Rac1 30 min but not 15 min after administration. The inhibition of hippocampal 5-HT2A receptors may induce the activation of Kalirin-7 and then activate Rac1, which needs to be investigated in future work.

The hippocampus is an important brain region involved in contextual fear memory, which receives dense serotonergic input from the raphe nuclei (Ihara et al., 1988). 5-HT is a modulatory neurotransmitter that plays a key role in the etiology of fear disorders (Bocchio et al., 2016). Dysregulation of the serotonergic system is a pathophysiological mechanism of stress-associated psychiatric disorders (Murrough et al., 2011). Several studies from our lab have shown that central 5-HT modulates anxiety and fear memory (Dai J. X. et al., 2008; Yu et al., 2015; Song et al., 2016). Central 5-HT deficient mice present less anxiety and enhancement of contextual fear memory (Dai J. X. et al., 2008). However, the present study showed that inhibition of hippocampal 5-HT2A receptors weakened but not enhanced contextual fear memory. The difference between these results may be interpreted as central 5-HT deficiency affects all types of 5-HT receptors and multiple brain areas in previous study, while this study only focused on the hippocampal 5-HT2A receptors. Cai et al. (2013) found that promoting accumulation of endogenous 5-HT potentiated fEPSPs in the CA1 area. Our previous study found that chronic administration of 5-HT reuptake inhibitor fluoxetine promoted the maturation of new-born neurons in the hippocampus (Jiang et al., 2014). These results indicated that activation of 5-HT system may enhance memory by modulating synaptic plasticity and new-born neurons. The present study showed that the activity of hippocampal 5-HT2A receptors modulated the spacing effect and Rac1 activity in contextual fear memory. Furthermore, we also demonstrated that 5-HT2A receptors interacted with Rac1. Our study provides a novel mechanism underlying the spacing effect in contextual fear conditioning. However, here we only investigated the regulation of 5-HT2A receptors altered hippocampal Rac1 activity in naïve rats but not in the contextual fear conditioning rats, which needs to be compared in future work.

## Conclusion

In summary, our study first demonstrates that activation of hippocampal 5-HT2A is a mechanism underlying the spacing effect, and forgetting related to molecular Rac1 is engaged in this process through interacting with 5-HT2A receptors. These results suggest that modulation of hippocampal 5-HT2A receptor-Rac1 pathway may be a promising therapeutic target for abnormal learning such as post-traumatic stress disorder (PTSD).

## Data Availability Statement

The datasets generated for this study are available on request to the corresponding author.

## Ethics Statement

The animal study was reviewed and approved by the ethics committee of Kunming Institute of Zoology.

## Author Contributions

This study was designed by LJ, RM and LX. Experiments in this study were performed by LJ, LW, CL, YY, MH, QZ and DY. The design and experiments of this study were supervised by LX and RM. LJ and RM revised the manuscript.

## Conflict of Interest

The authors declare that the research was conducted in the absence of any commercial or financial relationships that could be construed as a potential conflict of interest.
